# Supplementary material for: Tead1a Initiates Transcriptional Priming Through the TEAD1a/YAP‐Notch1‐Spi1/Cebpα Axis to Promote Neutrophil Fate
Source: Adv Sci (Weinh). 2025 Aug 28;12(41):e05441. doi: 10.1002/advs.202505441 (PMC12591127; doi:10.1002/advs.202505441)
Supplement: Supplementary file 4 — Supplementary Dataset [file ADVS-12-e05441-s004.zip › 3-2.zip!/3-2/02-2.cluster/cluster5/Enrich/ego_MF.pdf]

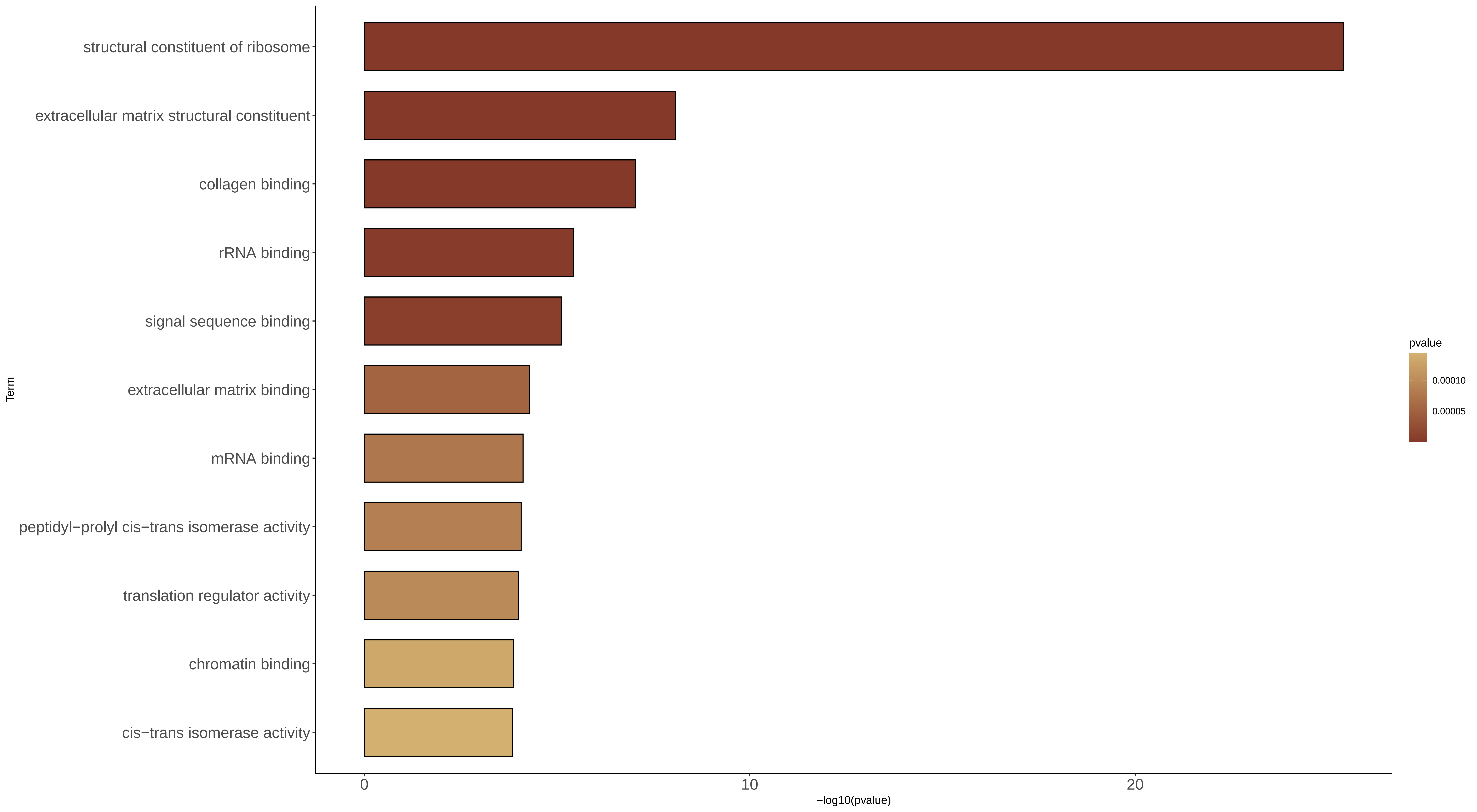
structural constituent of ribosome
extracellular matrix structural constituent
collagen binding
rRNA binding
signal sequence binding
extracellular matrix binding
mRNA binding
peptidyl−prolyl cis−trans isomerase activity
translation regulator activity
chromatin binding
cis−trans isomerase activity
Term
0
10
20
−log10(pvalue)
pvalue
0.00010
0.00005
